# Intentions in reconstructive plastic surgery among post-bariatric patients in Brazil’s public health service

**DOI:** 10.1590/acb405725

**Published:** 2025-08-08

**Authors:** Priscila Chiarello de Souza Pinto Abdalla, Juan Carlos Montano Pedroso, Rafael Cauê Katayama, Sarhan Sydney Saad, Gabriel de Almeida Arruda Felix, Marília Baeninger, Alfredo Gragnani

**Affiliations:** 1 Universidade Federal de São Paulo - Postgraduate Program in Translational Surgery - São Paulo (SP), Brazil.; 2 Universidade Federal de São Paulo - Postgraduate Program in Interdisciplinary Surgical Science - São Paulo (SP), Brazil.; 3 Universidade Federal de São Paulo - Department of Surgical Gastroenterology - São Paulo (SP), Brazil.; 4 Universidade Federal de São Paulo - Division of Plastic Surgery - São Paulo (SP), Brazil.

**Keywords:** Bariatric Surgery, Intention, Obesity, Morbid, Unified Health System

## Abstract

**Purpose:**

To evaluate post-bariatric patients’ intentions to undergo reconstructive plastic surgery in Brazil’s public health system.

**Methods:**

This cross-sectional study, conducted between May 2022 and February 2023, contacted 539 post-bariatric patients (≥ one year post surgery) via telephone, of whom 150 completed an online survey. Participants assessed their interest in reconstructive surgery and completed the validated Body-Q instrument to evaluate body image, sexual function, and related outcomes.

**Results:**

Most respondents were female (88%) and married (68%), with an average age of 43 years old. The preoperative and postoperative body mass indices were 44.5 and 28.1, respectively, with an average weight loss of 44 ± 12.8 kg. A total of 90.7% expressed interest in reconstructive surgery, particularly for the abdomen (75.3%) and breasts (68%). Lower education level was linked to higher aesthetic expectations (*p* = 0.014). Patients interested in arm surgery had significantly lower sexual function scores (*p* = 0.006). There was no significant correlation between total weight loss and surgical interest.

**Conclusion:**

Post-bariatric patients in Brazil’s public healthcare system showed a high demand for reconstructive surgery, with education level and body satisfaction influencing interest more than weight loss. Targeted counseling may help align expectations and enhance post-bariatric care for patients.

## Introduction

Obesity, characterized by excess body fat, is a significant global health challenge that affects millions of people and increases the risk of comorbidities, such as diabetes, hypertension, and cardiovascular diseases. According to the World Health Organization, obesity is defined by a body mass index (BMI) ≥ 30, with severe obesity (BMI ≥ 40) linked to markedly elevated health risks^1^. In Brazil, a 2019 survey by the Brazilian Institute of Geography and Statistics found that one in four adults is obese, emphasizing the scale of the problem^2^.

This global epidemic highlights the critical role of bariatric surgery as an effective intervention for sustained weight loss and improved health outcomes^3^. Despite its benefits, adherence to follow-up care after surgery remains a significant challenge, particularly in underserved populations^4^. Given the global context, this study narrows its focus to Brazil, where obesity rates have been escalating, and public health responses, including bariatric surgery, present unique challenges and opportunities.

Bariatric surgery was formally incorporated into Brazil’s Unified Health System in 2001. According to the Brazilian Society for Bariatric and Metabolic Surgery, this procedure is associated with a significant decrease in mortality rates and increased life expectancy for adults with severe obesity^5^. Bariatric surgery promotes significant weight loss and improves health and quality of life but often leaves excess skin, particularly in the abdomen, arms, and thighs, causing discomfort, infection, and body image dissatisfaction. The severity of skin laxity varies by factors such as age, skin quality, gender, and lifestyle, posing challenges for plastic surgeons and emphasizing the need for reconstructive surgery in post-obesity care^6^-^8^.

Despite its recognized benefits, not all post-bariatric patients pursue reconstructive surgery. Internationally, body contouring surgery rates vary widely, with cultural and healthcare disparities influencing patients’ intentions and access^9^-^14^. Studies from Finland, the Netherlands, China, and Chile indicate that only a minority of post-bariatric patients choose to undergo body contouring surgeries, and the type of preferential procedure performed also varies between countries. In Brazil, different factors, such as limited resources, cultural expectations, and patients’ wishes, may contribute to low uptake^13^-^16^. Patient-reported outcomes, measured using validated tools such as the Body-Q, have revealed that a higher BMI is associated with lower body image satisfaction. Body-Q, a validated patient-reported outcome tool, assesses the physical, psychological, and social impacts of body contouring treatments, offering nuanced insights into patient satisfaction and quality of life post-surgery^13^,^14^,^16^,^17^.

Few studies have explored post-bariatric surgery outcomes in low- and middle-income countries (LMICs), including Brazil, where healthcare systems and sociocultural factors differ significantly from those in high-income countries. Despite the rising prevalence of obesity and the inclusion of bariatric surgery in Brazil’s public health system, data on patient outcomes, such as health improvements and quality-of-life changes, are insufficiently documented, particularly in resource-constrained public healthcare settings. Addressing these gaps is critical to optimizing bariatric surgery for LMIC populations facing an escalating obesity crisis^18^-^20^.

Given the physical and psychological challenges associated with excess skin following bariatric surgery and the cultural and systemic factors affecting access to reconstructive procedures in Brazil, this study aimed to evaluate the intentions and expectations of Brazilian post-bariatric patients regarding reconstructive plastic surgery in the public health system. Given the cultural, clinical, and systemic disparities in Brazil’s Unified Health System, this study explored patients’ interest and expectations regarding reconstructive plastic surgery, rather than actual procedural rates.

## Methods

### Study design and setting

This primary observational cross-sectional analytic study was conducted at a Diadema State Hospital affiliated with a nonprofit medical association and was linked to the host institution. This study was approved by the Ethics Committee of the Universidade Federal de São Paulo. The study adhered to the American Association for Public Opinion Research for survey studies and the Strengthening the Reporting of Observational Studies in Epidemiology (STROBE) guidelines^21^,^22^.

### Participants

Between May 2022 and February 2023, 792 patients who had undergone bariatric surgery at least one year prior and had not undergone reconstructive surgery yet were contacted. Among them, 539 agreed to participate in the investigation and 150 completed the survey. Those who did not respond at first were contacted again at a different time as a reminder to do it. Patients were identified through hospital records and contacted via telephone, with up to three attempts for each. Participants were invited to complete an online survey (Google Forms). Eligible participants were required to be aged ≥ 18 years old, have no prior history of body contouring surgery, and consent to participate in the study. Informed consent was obtained from all participants prior to their involvement in the study, and no financial incentives or benefits were provided to ensure voluntary and unbiased participation in the study.

### Data collection

The survey consisted of 43 questions, including Body-Q scales and sociodemographic items developed following the recommendations of Chung et al.^23^, which contain a list of potential survey biases in question design, overall survey design, and survey administration. Estimated completion time was 15-20 minutes. Participants also completed the Body-Q instrument, a validated patient-reported outcome tool translated and validated into Brazilian Portuguese^24^. It evaluates the physical, psychological, and social impacts and sexual function of weight loss and body contouring treatments using scales that assess appearance concerns, physical discomfort due to excess skin, and quality of life. Excess skin was assessed using the “Appraisal of Excess Skin” subscale of the Body-Q questionnaire. Sexual function was also evaluated as part of the Body-Q. Informed consent was obtained from all participants before participation in the study. Responses were gathered electronically via Google Forms, with participants having the option to skip questions in accordance with the ethics committee recommendations.

### Data analysis

Data were analyzed using Statistical Package for the Social Sciences 26.0, and descriptive statistics were used to summarize the sociodemographic data and questionnaire responses. Independent t-tests were used to compare subgroup means, normality was assessed using the Kolmogorov-Smirnov test (*p* > 0.05), and significance was set at *p* < 0.05, with Bonferroni correction. Linear regression analyses were used to assess the effects of demographic and clinical variables (age, gender, marital status, education, and preoperative BMI) on body image, expectations, and sexual function using Rasch-transformed scales. Models tested overall fit, intercepts, and predictors with Type III Sum of Squares, reporting partial eta-squared for effect sizes and R-squared for model fit.

## Results

### Participant characteristics

Among the 792 patients contacted, 539 initially agreed to participate in the investigation, and 150 provided complete responses (response rate = 27.8%) (Table 1). Most respondents were female (88%), with an average age of 42.6 (standard deviation = 9.4) years old. Among the female participants, 68.1% were married, and 97.7% expressed interest in reconstructive plastic surgery. Similarly, 90.4% of the single women expressed interest in body-contouring surgery. Male participants accounted for 12% of the responses, with an average age of 47.2 (standard deviation = 10,9) years old. Among them, 53.3% expressed interest in surgery, and most were married (83.3%).

**Table 1:** Demographic and clinical profile of evaluated patients.

Variable	Description (n = 150)
**Age (years old), mean ± standard deviation**	43.2 ± 9.7
**Gender, n (%)**	
Female	132 (88)
Male	18 (12)
**Marital n (%)**	
Married	101 (67.3)
Single	32 (21.3)
Divorced	11 (7.3)
Common-law partners	4 (2.6)
Widowed	2 (1.3)
**Education level, n (%)**	
Complete primary education	2 (1.3)
Incomplete secondary education	8 (5.3)
Complete secondary education	53 (35.3)
Incomplete higher education	16 (10.7)
Complete higher education	58 (38.7)
Postgraduate	13 (8.7)

### Educational background and expectations

Most participants had completed higher education (38.7%), followed by high-school graduates (35.3%) and postgraduates (8.7%) (Table 2). Lower educational levels included incomplete higher education (10.7%), incomplete high school (5.3%), and elementary school (1.3%) education. Participants with lower education levels tended to expect more transformative outcomes from surgery (*p* = 0.014), whereas those with higher education levels exhibited more balanced expectations (Table 3).

**Table 2:** Participants’ education profile.

Comparison*	Mean difference	Standard error	P-value	95%CI
Elementary or incomplete high school - complete high school or incomplete higher education	9.29	7.08	> 0.999	-9.68-28.26
Elementary or incomplete high school - complete higher education	17.91	7.20	0.085	-1.39-37.20
Elementary or incomplete high school - postgraduate	19.38	8.64	0.159	-3.75-42.50
Complete high school or incomplete higher education - complete higher education	8.61	3.55	0.099	-0.89-18.11
Complete high school or incomplete higher education - postgraduate	10.08	5.94	0.551	-5.82-25.99
Complete higher education - postgraduate	1.47	6.08	> 0.999	-14.82-17.76

95%CI: 95% confidence interval;

* Bonferroni multiple comparisons.

**Table 3:** Patients’ expectations for surgical results (n = 150).

I will look wonderful after surgery	N	%
Strongly agree	61	40.7
Slightly agree	53	35.3
Slightly disagree	20	13.3
Strongly disagree	4	2.7
No response	12	8.0
**I will be transformed after surgery**	**N**	**%**
Strongly agree	77	51.3
Slightly agree	40	26.7
Slightly disagree	15	10.0
Strongly disagree	6	4.0
No response	12	8.0

### Weight loss and body mass index

**Figure 1:**
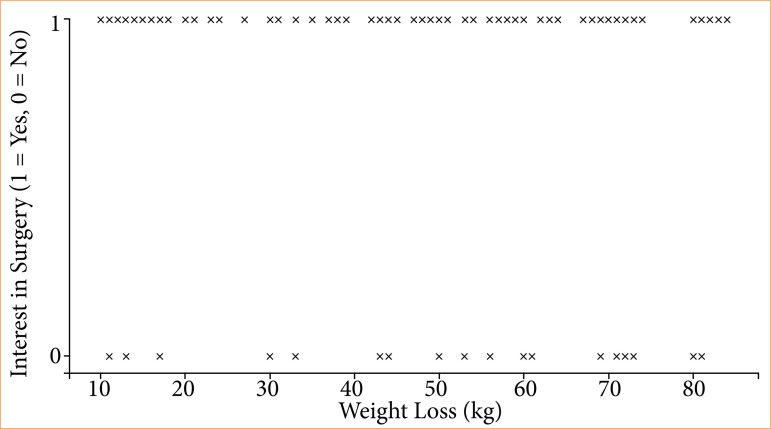
Correlation between weight loss and desire for reconstructive plastic surgery.

Source: Elaborated by the authors.

The mean preoperative BMI was 44.5 kg/m^2^ (standard deviation ± 6.3) (severe obesity, grade III), which decreased to 28.1 kg/m^2^ (standard deviation ±4.4) (overweight) postoperatively. Participants reported weight loss ranging from 11 to 80 kg, with an average loss of 44 kg (standard deviation ± 12.8) (Table 4). Despite significant weight loss, 62% of respondents reported dissatisfaction with their body image, as assessed using the Body-Q questionnaire. As shown in Fig. 1, there was no clear correlation between weight loss and the desire to undergo reconstructive surgery. Patients reporting both high and low weight-loss levels were distributed across both groups.

**Table 4:** Participants’ physical attributes profile.

Variable	Description (n = 150)
Current weight (kg), mean ± SD	76.2 ± 14.7
Weight loss (kg), mean ± SD	44 ± 12.8
Pre-operative BMI (kg/m2), mean ± SD	44.5 ± 6.3
Post-operative BMI (kg/m2), mean ± SD	28.1 ± 4.4
BMI loss (%), mean ± SD	36.4 ± 7.6
Considers undergoing reconstructive plastic surgery?, N (%)	
No	14 (9.3)
Yes	136 (90.7)
Areas considered for surgery, n (%)	
Arms	58 (38.7)
Thighs	36 (37.3)
Breasts	104 (69.3)
Abdomen	115 (76.7)
Others	18 (12.0)

SD: standard deviation; BMI: body mass index.

### Surgical interests

Overall, 90.7% of participants expressed an interest in reconstructive plastic surgery. The most frequently desired procedures targeted the abdomen (75.3%) and breasts (69.3%), followed by the arms (38.7%) and thighs (37.3%) (Table 4). Other areas of interest included back (4%), neck (3.3%), buttocks (3.3%), and armpits (1.3%). Among the participants who were not interested in abdominal surgery, breast surgery was the most frequently preferred alternative (Table 5).

**Table 5:** Surgical regions preferred by patients not interested in abdominal surgery.

Body region	Description (n = 35)
Does not consider undergoing reconstructive plastic surgery	14 (40٪)
Arms	10 (28.6٪)
Thighs	3 (8.6%)
Breasts	16 (45.7٪)
Others	3 (8.6٪)

### Sexual function and body image

Participants interested in arm surgery reported significantly lower average sexual function scores (*p* = 0.006). Lower scores were also observed in those who desired surgery in other body areas (*p* = 0.022) (Table 6). These findings aligned with the broader dissatisfaction with body image among the cohort, despite substantial weight loss.

**Table 6:** Sexual function score by desired surgical area*.

Body regions patient wants to operate	Sexual function score (mean ± standard deviation)	n	*p* -value
Arms			**0.006**
No	50.2 ± 17.7	78	
Yes	41 ± 20.6	58	
Thighs			0.451
No	47.3 ± 18	80	
Yes	44.8 ± 21.5	56	
Breasts			0.089
No	51.4 ± 21	32	
Yes	44.7 ± 18.8	104	
Abdomen			0.609
No	48.3 ± 15.6	21	
Yes	45.9 ± 20.1	115	
Others			**0.022**
No	47.8 ± 18.8	118	
Yes	36.5 ± 21.4	18	

* Independent t-test.

### Linear regression models

Regression analyses revealed that the overall model for body image was not statistically significant (*p* = 0.493, R-squared = 0.033). For body expectations, the model was not statistically significant (*p* = 0.055, R-squared = 0.079), with education level (*p* = 0.005) as the only significant predictor of body expectations. For sexual function, the regression model was significant (*p* < 0.001, R-squared = 0.152), with several predictors showing significant effects. Gender (B = 12.321, *p* = 0.029), marital status (B = -6.176, *p* = 0.002), education level (B = 2.300, *p* = 0.019), and preoperative BMI (B = 0.519, *p* = 0.047) were significant predictors. The Partial Eta Squared values of the model indicated that gender, marital status, and education level had medium- to large-effect sizes. However, age was not a significant predictor (*p* > 0.05). These findings are correlational and do not imply causation.

## Discussion

Our findings highlighted the stark demand for reconstructive surgery after bariatric surgery, aligning with and extending the current understanding of post-surgical patient needs. Bariatric surgery has shown significant benefits in improving the quality of life and reducing the comorbidities associated with obesity. However, post-bariatric patients often face challenges due to excess skin, which negatively affects their physical and psychological well-being^16^,^25^. Previous research emphasized that addressing these issues through reconstructive surgeries not only enhances functionality but also psychological satisfaction^3^.

A recent study in a Brazilian post-bariatric population indicated that 90% of patients experienced considerable discomfort due to excess skin, and over 60% expressed dissatisfaction with their body image^26^.

Many patients feel disappointed to find that despite losing weight, they are unable to wear certain clothing because of excess skin. Although patients are typically advised of this, some assume their skin will retract on its own and are unprepared for this adverse effect of weight loss^8^.

The Body-Q tool provides objective data on body image, aiming to minimize subjective biases often inherent in self-perception^27^. Furthermore, the standardized scoring system prevents the researcher’s own views or subjectivity from influencing the interpretation of the results, prioritizing scientific rigor.

Several factors can influence the response rate of a survey, with higher response rates generally reflecting more representative and reliable sample. Online surveys may offer advantages over traditional methods by reaching larger audiences at lower costs. However, contacting participants through digital means may result in lower participation compared to more conventional approaches^28^.

The findings of this study highlight demographic factors, such as education, gender, marital status, and previous BMI experience, significantly influenced body expectations and sexual function, whereas body image was not significantly predicted by these variables. The impact of education on body expectations suggests a potential role for cognitive factors in shaping body perceptions, whereas the significant effects on sexual function emphasize the importance of considering gender, marital status, and clinical experiences to improve sexual health outcomes. Notably, the lack of correlation between total weight loss and the desire for surgery points out to the multifactorial nature of surgical interest. Factors such as body image, education, and cultural expectations may play a more prominent role than weight metrics by themselves. These results underscore the complexity of body image and sexual function, suggesting that additional psychological and emotional factors may be important for understanding and addressing these constructs in clinical practice. Further research is needed to explore these factors and refine interventions.

Sexual function was an important outcome in this study. Patients interested in arm surgery had lower Body-Q sexual function scores. Arm-related concerns may disproportionately affect sexual function outcomes because of their visibility and impact on body image during intimate interactions. Unlike the breasts or thighs, which may be more concealed, excess skin on the arms can be a constant visual reminder of dissatisfaction, particularly during activities that involve gestures or physical closeness. Studies using the Body-Q instrument have shown that dissatisfaction with visible areas of the body, such as the arms, significantly correlates with lower sexual function scores^8^,^16^. This can stem from heightened self-consciousness and reduced confidence during intimate encounters.

Breast and thigh concerns, while impactful, may primarily affect aspects of body image or physical discomfort, rather than sexual function. For example, dissatisfaction with breasts often ties aesthetic expectations with femininity rather than direct sexual function^29^. Thigh concerns, meanwhile, may predominantly relate to mobility and comfort^3^.

Our findings aligned with international trends, revealing a high demand for body contouring surgery among post-bariatric patients, despite cultural and systemic barriers in Brazil’s public healthcare system. While aesthetic motivations drive demand in Western countries, cultural norms in parts of Asia favor modesty, resulting in a lower uptake of elective surgeries^13^,^16^. However, in Brazil, the high demand contrasts sharply with the limited access to public healthcare, where the Unified Health System prioritizes medically necessary procedures over aesthetic ones. This disparity is comparable to systems like the Netherlands, where reimbursement requires demonstrated medical necessity^14^.

Studies in Europe and North America have shown that body contouring surgeries improve psychological well-being, body satisfaction, and self-esteem^8^,^30^. Our results reflect this trend, with 90.7% of participants expressing an interest in reconstructive plastic surgery, particularly for the abdomen and breast. However, financial and systemic constraints prevent many Brazilian patients from accessing these benefits, underscoring the need for policy changes to support comprehensive post-bariatric care.

## Educational background and expectations

This study also revealed important sociodemographic patterns. While most participants had higher educational levels (38.7%), those with lower educational attainment exhibited elevated aesthetic expectations. This finding suggests that differences in educational background may influence patients’ understanding of surgical outcomes and risks of surgery. Transformative expectations were reported by 78% of participants, placing considerable pressure on plastic surgeons to manage these expectations effectively^31^.

The abdomen is the most cited area of interest in surgery. In Brazil’s public health system, abdominal surgery is approved as a reconstructive procedure, described as dermolipectomy, and is considered reconstructive (addressing overhanging abdominal skin, rectus abdominis muscle diastasis, and potential hernias). The possibility that this procedure might be government-funded may subconsciously influence patients’ preferences, introducing potential bias.

Breast surgery was the second most frequently cited area, with 68% of participants (respondents could choose more than one area of the body), which only some studies in the literature have mentioned^29^,^32^. This result may stem from several factors, including the predominance of female participants, the cultural significance of breasts in female sexuality, and cultural differences across countries.

This study revealed a direct correlation between sexual satisfaction, body satisfaction, and percentage loss in BMI. Furthermore, lower body image scores and significant weight loss have increased the interest in plastic surgery.

Regarding patient expectations for reconstructive surgery, 78% anticipated transformative results, which places considerable pressure on plastic surgeons, given the potential for unrealistic expectations. The study observed that patients with lower educational levels tended to have higher expectations, possibly because of challenges in communication and understanding during medical consultations^31^. This underscores the need for well-prepared surgeons to manage this patient profile. It is essential for patients to understand that these procedures, following massive weight loss, are complex and should not be compared with conventional cosmetic surgeries^33^.

Scarring is another important consideration in body contouring. Many patients are unaware of the extent of their disease, underscoring the need for clear communication tailored to each patient’s educational level. This approach can help manage expectations, reduce dissatisfaction, and address potential medicolegal concerns^34^-^36^.

## Gender and cultural differences

Gender differences were notable, with female participants expressing significantly higher interest in surgery (97.7% of married women and 90.4% of single women) than male participants (53.3%). These findings aligned with cultural norms emphasizing female aesthetics, particularly in Brazil, where societal expectations may heighten the desire for surgical intervention. Abdominal and breast surgeries were the most desired procedures among women, whereas men exhibited less pronounced preferences for these procedures.

## Systemic barriers in Brazil’s public healthcare system

Brazil’s Unified Health System provides limited access to reconstructive plastic surgery and prioritizes procedures deemed medically necessary, such as dermolipectomy for overhanging abdominal skin. The availability of public funding for these surgeries may subconsciously bias patient preference, particularly for abdominal procedures^37^,^38^. However, the high demand for breast surgeries and other body areas highlights unmet needs that extend beyond the scope of current public health policies.

## Implications for patient care

This study underscores the need for tailored preoperative counseling to address unrealistic expectations, particularly in patients with lower educational levels^35^,^39^. Clear communication about achievable outcomes, the complexity of reconstructive surgeries, and the potential for scarring is critical for reducing postoperative dissatisfaction and mitigating medicolegal risks36. Additionally, the association between body image dissatisfaction and reduced sexual function highlights the importance of integrating psychological support into post-bariatric care^33^.

## Limitations and strengths

This study have some limitations. Reliance on self-reported data introduces the possibility of social desirability bias, particularly regarding sensitive topics such as body image and sexual satisfaction. This bias may have led participants to underreport their dissatisfaction or overstate their intention to undergo surgery. Furthermore, the online survey format may have excluded individuals with limited internet access or low digital literacy, potentially skewing the sample toward younger, more educated, or more tech-savvy participants. The response rate of 27.8% also raises concerns about representativeness, as it may not fully capture the diversity of experiences among the target population. Low response rates can be an indicative of systemic barriers that disproportionately affect minority and underserved populations^4^. This study did not track whether patients ultimately underwent reconstructive surgery, which limits the conclusions about real-world procedural uptake; however, this was beyond the scope of the current study. Future longitudinal studies are warranted.

The cross-sectional design of this study limits the ability to establish causal relationships between variables, such as the impact of BMI changes on body satisfaction and surgical intentions. Additionally, the single-center setting may restrict the generalizability of the findings to other regions of Brazil or healthcare systems with differing structures and resources. Furthermore, the use of a non-validated questionnaire developed by the authors to assess patient intentions represents a significant limitation, as it may affect the reliability and reproducibility of the findings. Additionally, reliance on self-reported data introduces the possibility of recall or social desirability bias, which could influence response accuracy. Demographic data of participants who did not complete the survey were not collected. However, this is an important area for future studies to address, as it could provide further insights into non-response bias and the representativeness of our sample.

Despite its limitations, this study provides valuable insights into the often underrepresented population of post-bariatric patients in Brazil’s public healthcare system. By utilizing the validated Body-Q instrument, subjective biases are minimized, and body image and satisfaction are comprehensively assessed, aligning with international standards. The relatively large sample size (792 eligible patients, 150 complete responses) enabled a meaningful analysis of the sociodemographic and clinical factors influencing surgical intentions, while its focus on Brazil’s Unified Health System offers insights for targeted policy changes. Future research should enhance generalizability by using diverse samples, incorporating qualitative methods, and conducting long-term studies to examine the impact of reconstructive surgery on the quality of life and mental health. Educational interventions for patients and healthcare professionals could further align expectations and improve decision making.

## Conclusion

This study underscores the high demand for reconstructive plastic surgery among post-bariatric patients, particularly for abdominal (75.3%) and breast (68%) surgeries. The analysis also revealed that patients with lower education levels tended to have higher expectations for aesthetic outcomes, despite most respondents having completed higher education.

Effective management of post-bariatric patients requires careful guidance to set realistic expectations and reduce potential postoperative dissatisfaction. Clear communication about achievable outcomes is essential to support these patients’ well-being and strengthen the case for SUS and health insurance providers to approve these procedures, emphasizing their functional and psychological benefits.

## Data Availability

The data will be available upon request.
